# A novel automated image analysis system using deep convolutional neural networks can assist to differentiate MDS and AA

**DOI:** 10.1038/s41598-019-49942-z

**Published:** 2019-09-16

**Authors:** Konobu Kimura, Yoko Tabe, Tomohiko Ai, Ikki Takehara, Hiroshi Fukuda, Hiromizu Takahashi, Toshio Naito, Norio Komatsu, Kinya Uchihashi, Akimichi Ohsaka

**Affiliations:** 1 Department of Next Generation Hematology Laboratory Medicine, Juntendo University Graduate School of Medicine, Tokyo, Japan; 2 Sysmex Corporation, Kobe, Japan; 3 Department of Clinical Laboratory Medicine, Juntendo University Graduate School of Medicine, Tokyo, Japan; 4 Department of General Medicine, Juntendo University Graduate School of Medicine, Tokyo, Japan; 5 Department of Hematology, Juntendo University Graduate School of Medicine, Tokyo, Japan; 6 Department of Transfusion Medicine and Stem Cell Regulation, Juntendo University Graduate School of Medicine, Tokyo, Japan

**Keywords:** Myelodysplastic syndrome, Diagnostic markers

## Abstract

Detection of dysmorphic cells in peripheral blood (PB) smears is essential in diagnostic screening of hematological diseases. Myelodysplastic syndromes (MDS) are hematopoietic neoplasms characterized by dysplastic and ineffective hematopoiesis, which diagnosis is mainly based on morphological findings of PB and bone marrow. We developed an automated diagnostic support system of MDS by combining an automated blood cell image-recognition system using a deep learning system (DLS) powered by convolutional neural networks (CNNs) with a decision-making system using extreme gradient boosting (XGBoost). The DLS of blood cell image-recognition has been trained using datasets consisting of 695,030 blood cell images taken from 3,261 PB smears including hematopoietic malignancies. The DLS simultaneously classified 17 blood cell types and 97 morphological features of such cells with >93.5% sensitivity and >96.0% specificity. The automated MDS diagnostic system successfully differentiated MDS from aplastic anemia (AA) with high accuracy; 96.2% of sensitivity and 100% of specificity (AUC 0.990). This is the first CNN-based automated initial diagnostic system for MDS using PB smears, which is applicable to develop new automated diagnostic systems for various hematological disorders.

## Introduction

Myelodysplastic syndromes (MDS) are heterogeneous clonal hematopoietic stem cell disorders characterized by ineffective and neoplastic hematopoiesis and dysplasia of one or more of the major hematopoietic lineages associated with a variable risk of later acute leukemia^1–3^. Although several susceptibility genes have recently been identified (e.g., *DNMT3A*, *TET2*, *ASXL1*, *TP53*, and *RUNX1*), the pathogenesis is not yet completely understood^4,5^. Therefore, diagnostic workup relies on conventional tests including a complete blood count (CBC), morphological examinations of PB smear and bone marrow (BM) aspiration and biopsy, and flow cytometry^6,7^. The first two tests are valuable initial diagnostic steps, being much less invasive and costly than the other examinations.

Currently, normal leukocytes can be differentiated using automated hematology analyzers equipped with optical sensors and mathematical computer-based algorithms (e.g., the Sysmex XN series)^8,9^. However, because the morphology of dysplastic blood cells in patients with hematological disorders is much more elaborate than that of normal cells, manual microscopic examinations remain the mainstay of diagnosis, which are time-consuming, demanding, and subjective. Thus, many industrial and academic researchers have sought to develop efficient and accurate automated diagnostic systems. Current advances in computer technology have been used to derive automated diagnostic systems for leukemia. Over the past decade, more than 20 studies have attempted to diagnose of hematological malignancies mainly acute lymphoblastic leukemia (ALL) using various mathematical algorithms to recognize and classify cell images^10–12^. This process requires several complex steps such as preprocessing, segmentation, feature extraction, and classification^13^. Recently, convolutional neural networks (CNNs), advanced forms of deep learning, have been used to optimize the parameters automatically, without the need for mathematical algorithms^14^. CNNs classify cell images more accurately than conventional neural networks or machine-learning systems^14^.

In this study, we first developed an automated blood cell image-recognition system using a deep learning system (DLS) powered by CNNs that simultaneously classifies 17 blood cell types and 97 morphological features of such cells. Second, we created an automated MDS diagnostic support system by combining the CNN-based image-recognition system with a form of extreme gradient boosting (XGBoost). Then, we evaluated the diagnostic system using the PB smear samples obtained from patients with MDS or aplastic anemia (AA). We chose AA for the comparison because dysmorphic cells are not often evident in PB samples of AA compared to MDS although both diseases are characterized by reticulocytopenic anemia, variable neutropenia and thrombocytopenia due to BM failure^15^. Our diagnostic system successfully differentiated MDS from AA with high accuracy compared to human diagnoses. Here, we described the details of how we developed this new diagnostic system of MDS.

## Results

### Performance of the DLS in terms of morphological classification of blood cell types

**Figure 1:**
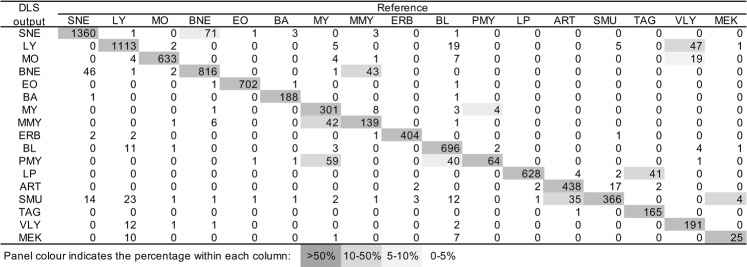
Confusion matrix for the 17 types of differentiated blood cells. The 17 blood cell types differentiations by DLS are compared to the reference classification of validation dataset. SNE, segmented neutrophil; LY, lymphocyte; MO, Monocyte; BNE, band neutrophil; EO, eosinophil; BA, basophil; MY, myelocye; MMY, Metamyelocyte; ERB, erythroblast; BL, blast; PMY, promyelocyte; LP, large platelet; ART, artifact; SMU, smudge; TAG, thrombocyte aggregation; VLY, variant lymphocyte; MEK, megakaryocyte.

The DLS performance in terms of morphological classification of blood cell types was validated using the validation datasets generated as described in Material and Method (Table 1). Table 2 shows that the DLS cell differentiation sensitivity ranged from 93.9 to 99.8%, and the specificity from 96.0 to 100%. We compared the DLS performance with that of the DI-60, a conventional computer-based image-recognition system of automated hematology analyzer (Sysmex), and observed that the DLS was more sensitive and specific (Supplemental Table S1). Figure 1 shows the DLS confusion matrix for the 17 blood cell types, compared to the reference classification of validation dataset. The DLS tended to misclassify segmented neutrophils as band neutrophils, lymphocytes as variant lymphocytes, band neutrophils as meta-myelocytes, meta-myelocytes as myelocytes, promyelocytes as myelocytes, and large platelets as thrombocyte aggregations.

**Table 1:** Images in the datasets.

Cell type	images for training	images for validation
Segmented Neutrophil	315,777	1,432
Band Neutrophil	19,191	896
Metamyelocyte	2,235	196
Myelocyte	4,596	418
Promyelocyte	699	70
Blast	11,237	790
Lymphocyte	149,524	1,177
Variant Lymphocyte	4,521	262
Monocyte	36,734	641
Eosinophil	16,186	705
Basophil	2,808	205
Large Platelet	62,985	631
Megakaryocyte	453	31
Platelet Aggregation	2,541	208
Erythroblast	4,823	409
Smudge	54,027	391
Artifact	6,693	478

**Table 2:** Cell classification performance of the DLS.

Cell type	Sensitivity (%)	Specificity (%)
Segmented Neutrophil	98.0	97.7
Band Neutrophil	98.0	97.0
Metamyelocyte	93.9	96.0
Myelocyte	98.1	96.9
Promyelocyte	98.6	97.6
Blast	97.2	98.7
Lymphocyte	99.3	96.5
Variant Lymphocyte	95.0	98.2
Monocyte	99.5	99.1
Eosinophil	99.6	100.0
Basophil	98.5	99.5
Large Platelet	99.7	99.4
Megakaryocyte	93.5	99.6
Platelet Aggregation	95.7	99.3
Erythroblast	99.8	99.4
Smudge	95.4	98.0
Artifact	99.0	98.7

**Figure 2:**
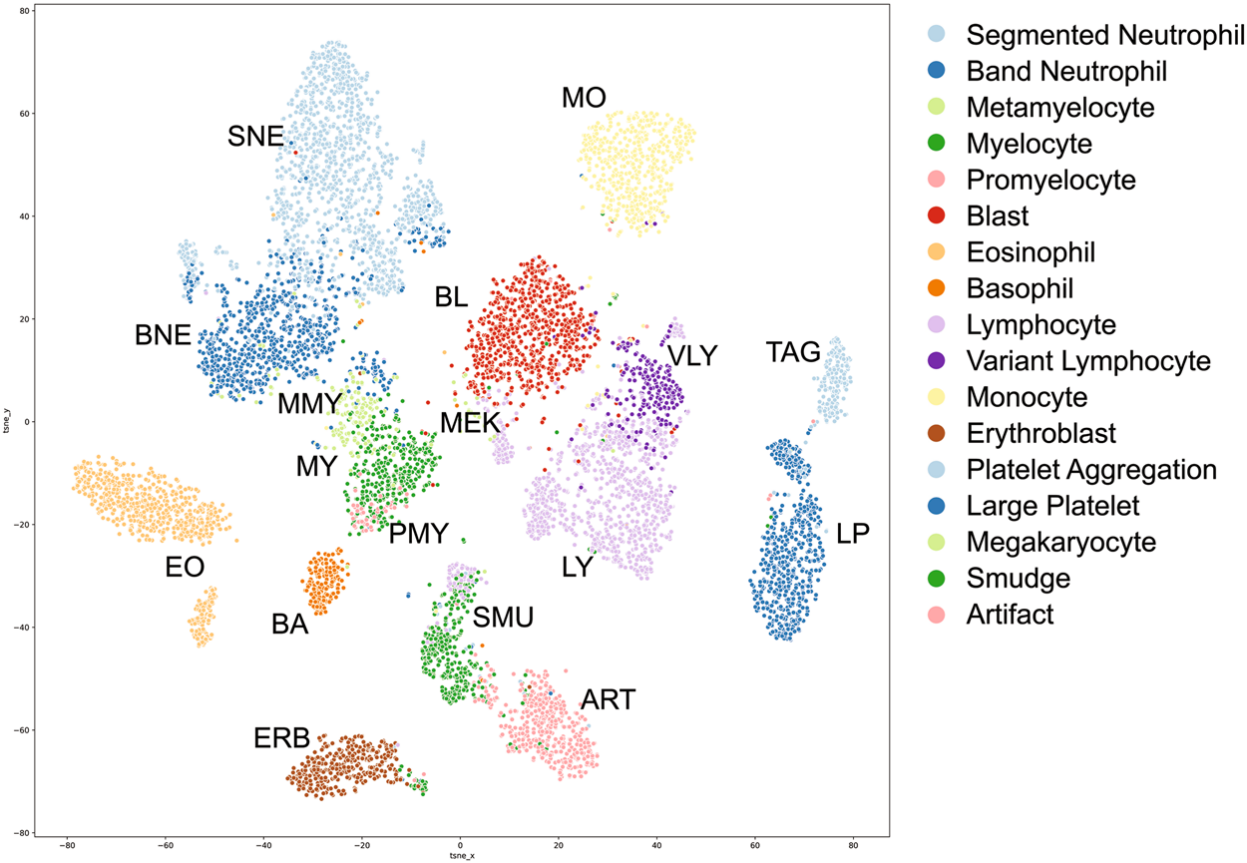
t-SNE visualization of the “DLS last hidden layer” representations of 17 blood cell types. The 17 types of cell clusters are colored and labeled with abbreviations of the cell types (inset).

To dissect such misclassifications in the confusion matrixes, we examined the internal features learned by the DLS using t-distributed Stochastic Neighbor Embedding (t-SNE)^16^. Figure 2 shows cell images projected from the 2,048-dimensional output of the last hidden layer of the DLS onto two dimensions. Blasts (red dots) remain in the center of the field. Three types of cells (granulocytes, lymphocytes, and monocytes) surround the blasts. Granulocytes are distributed to the left of the blasts all the way from the most differentiated segmented neutrophils (top) to the most premature promyelocytes (bottom). On the contrary, lymphocytes are located to the right of the blasts, and are distributed from premature variant lymphocytes (top) to mature lymphocytes (bottom). Eosinophils, basophils, and monocytes are found in relatively discrete locations. Some band neutrophils lie within metamyelocytes. The DLS may thus be unable to differentiate these two cell types. Megakaryocytes lie adjacent to blasts, which might compromise the accuracy of image recognition. Large platelets and platelet aggregations lie at the extreme right of the field.

### DLS performance in terms of recognizing morphological abnormalities

Next, we explored how accurately the DLS automatically detected dysmorphic features of peripheral blood cells of each hematopoietic lineage in the validation datasets generated as described in Material and Method. Table 3 shows the sensitivity, specificity, and area under the curve (AUC) calculated from the Receiver Operatorating Characteristic (ROC) curve. The sensitivity was high (80 to 98%) except for detection of giant platelets. Representative images of dysmorphic peripheral blood cells in the validation datasets are shown in Supplementary Fig. 1.

**Table 3:** Classification performance of our system in terms of dysmorphic blood cells.

Cell type	Sensitivity (%)	Specificity (%)	AUC	images for validation*
**Blast**				
abnormal shape of nuclei	95.3	94.4	0.975	107
abnormal granulation	85.5	95.4	0.926	62
vacuoles	92	94.5	0.950	112
**Neutrophil**				
Pelger-Huet anomaly	93.4	95.9	0.960	61
spherical /ovoid nucleus	96.2	97.4	0.978	53
hypersegmentation	81.3	97.5	0.904	64
degranulation	86.2	92.6	0.955	130
abnormal granulation	98.3	92.2	0.986	173
giant	91.8	93.9	0.977	233
vacuoles	81.9	93.8	0.931	105
toxic granulations	95.9	97.3	0.992	244
Döhle body	90.5	86.6	0.947	84
**Lymphocyte**				
cleaved nuclei	93.5	95.2	0.969	139
increased N:C ratio	94.7	95	0.966	75
abnormal chromatin	92.2	94.7	0.958	90
abnormal shape of nuclei	92.9	92.8	0.953	113
granular lymphocyte	82.5	94.5	0.903	154
vacuoles	81.8	92.5	0.893	110
**Erythroblast**				
irregular shape	80	59.9	0.878	55
**Large platelet**				
giant platelet	61.5	97.5	0.801	174

*dysmorphic cell types with more than 30 images for validation were shown.

### DLS performance in terms of the differential diagnosis of MDS and AA

Although both MDS and AA can trigger pancytopenia, dysmorphic blood cells are not often evident in AA in contrast to MDS^17^. In MDS, neutrophils undergo degranulation or abnormal granulation and may exhibit the pseudo-Pelger-Huet anomaly and/or hypo- or hyper-segmentation; giant neutrophils and platelets are evident^17,18^. To allow automated diagnosis of MDS, 114 image-pattern parameters from smears of MDS and AA patients were fed to XGBoost, which automatically analyzed the extent and nature of normal and dysmorphic images, and then diagnosed MDS or non-MDS using the test datasets.

**Figure 3:**
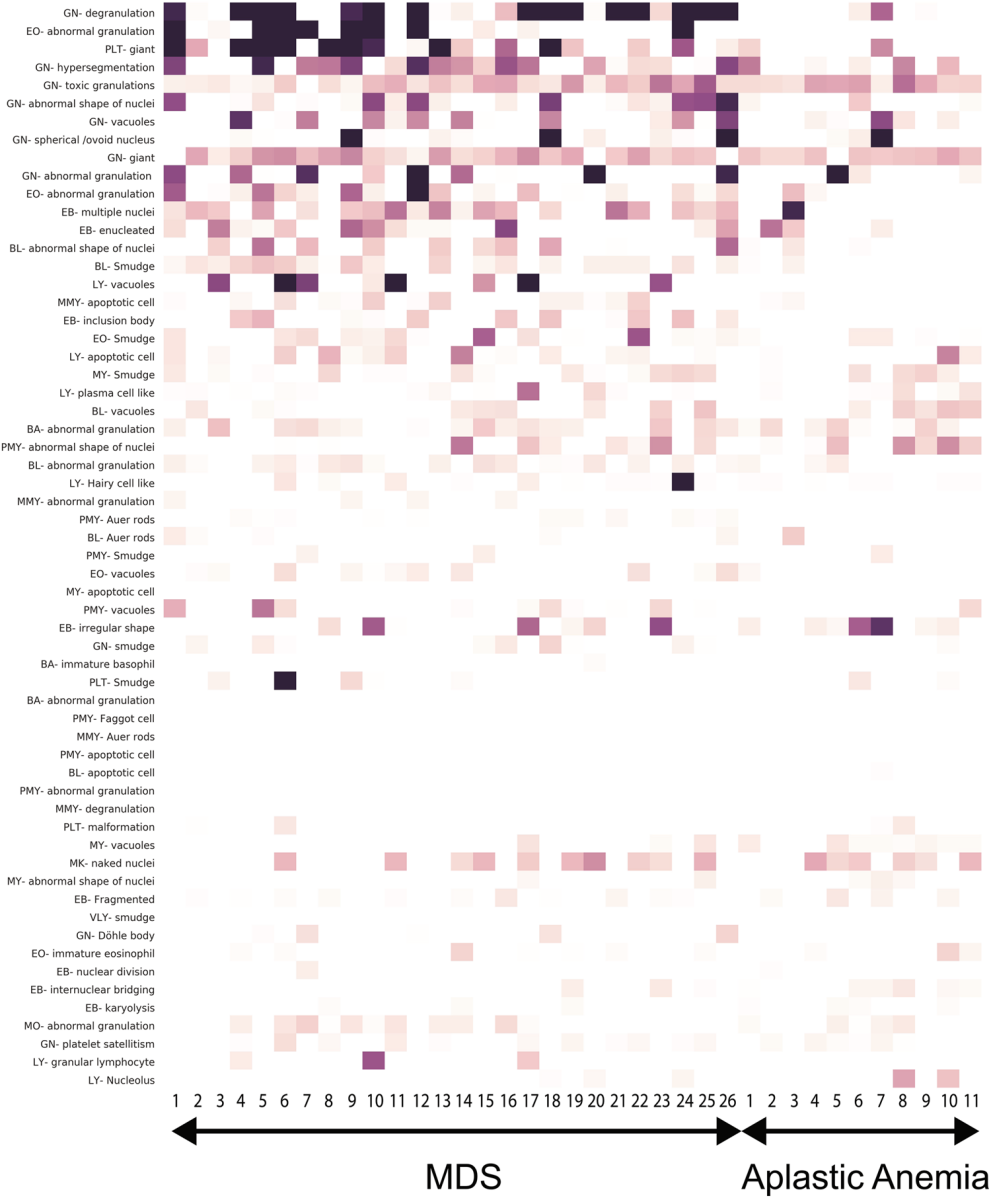
A heat map indicating the extent of blood cell dysmorphic features. Each row indicates the dysmorphic features of cells of a single case (MDS cases 1–26 and AA cases 1–11). SHAP values were calculated and used to generate the heat map. GN, neutrophils; LY, lymphocytes; EO, eosinophils; BA, basophils; MY, myelocytes; MMY, metamyelocytes; EB, erythroblasts; BL, blasts; PMY, promyelocytes; PLT, large platelets; VLY, variant lymphocytes; MK, megakaryocytes.

Figure 3 shows a heat map of dysmorphic cell features based on the SHapley Additive exPlanations (SHAP) values analyzed by our system for each case (MDS: 1–26; AA: 1–11 cases). The darker the color, the more dysmorphic the cells. The rates of detection in MDS samples of abnormal neutrophil degranulation and the pseudo-Pelger-Huet anomaly, and giant platelets, were significantly higher than in AA samples. However, dysmorphic features of lymphocytes, basophils, eosinophils, and promyelocytes did not assist differentiation of MDS from AA, consistent with the diagnostic features of MDS evident in BM aspirates^15^.

**Figure 4:**
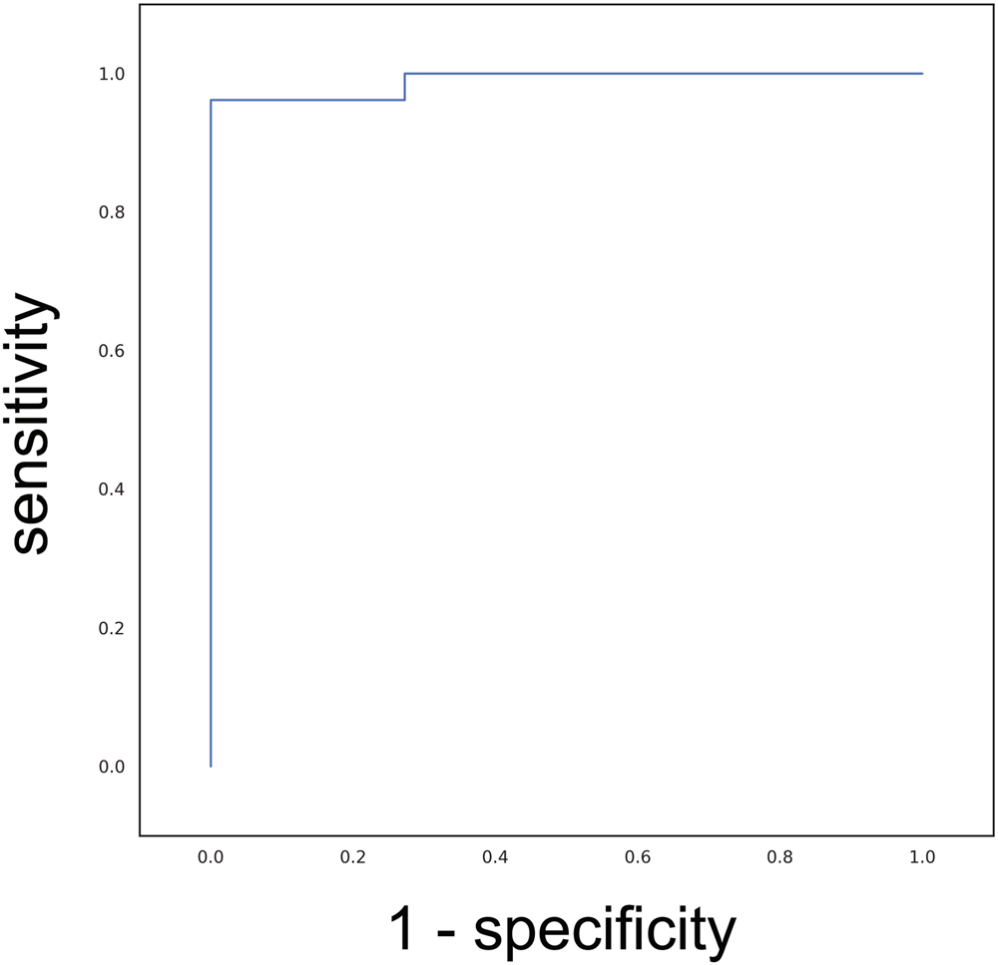
Differential diagnostic performance of the DLS. The AUC was used to measure performance; the maximum value is 1.

The sensitivity and specificity of the DLS performance in terms of the differential diagnosis of MDS and AA were 96.2 and 100%, respectively. The AUC of the ROC curve was 0.990 (Fig. 4).

## Discussion

We developed a novel MDS diagnostic support system using PB smears. The system featured a CNN-based image recognition DLS and an EGB-based decision-making algorithm, XGBoost.

The conventional computer running image-recognition system engage in algorithms for preprocessing, segmentation, feature extraction, and classification, which are similar to how images are recognized by the human eye. In these systems, many mathematical algorithms are used: (1) histogram equalization, Gaussian filtering, or median filtering for preprocessing; (2) K-means clustering or calculation of Fuzzy C-means for segmentation; (3) geometrical or shape features for feature extraction; and (4) support vector machines (SVMs), artificial neural networks, or random forests (RFs)^13^ for classification. However, optimization of these parameters are not straightforward because the variety of format, scaling and bit-size of algorithms and the difficulties of parameter tuning, which trigger communication mismatches between algorithms. On the contrary, neural networks perform all of these complicated tasks simultaneously, and there is no explicit need for complex mathematical models. Neural networks consist of piles of layers. Each layer is analogous to a neuron of the brain.

Recently, deep CNNs featured five convolutional layers were employed to detect ALL cells and to classify into three morphological subtypes (i.e., L1, L2, and L3, French-American-British Classification), and achieved 95–99% of sensitivity and specificity^10^. The performance of this CNN was superior than the ones of previous studies using mathematical algorithms such as support vector machines, the K nearest-neighbor approach, and hybrid hierarchial classifiers^12,19^.

Detection and classification of myeloid malignant cells including MDS requires capability to differentiate normal and abnormal morphological features in three hematopoietic lineages including myeloid cells, erythroblasts and platelets in PB smears. Therefore our CNN-based image recognition DLS featured eight convolutional layers in total to detect and classify more complicated images than the ones of ALL. Finally, our system recognized over 100 patterns in cell size and cytoplasmic morphological features, and achieved >90% sensitivity and specificity in the diagnosis of MDS compared to the human eye. But why not 100%? As shown in the t-SNE plots (Fig. 2), it might be very difficult to differentiate the cells that are continuously differentiating in a same lineage. For example, even by human eyes, it is difficult to distinctly differentiate band neutrophils from less matured metamyelocytes. However, further training may improve the DLS accuracy more effectively than human eyes with higher reproducibility.

We, then, created an MDS diagnostic support system featuring a highly trained cell image-recognition system combined with a decision-making algorithm based on XGBoost, which afforded 96% sensitivity and 100% specificity in terms of differential diagnosis of MDS and AA. These results were consistent with the recently developed automated diagnostic system of dermatological disease based on well-trained CNNs which demonstrated the comparable performance to human diagnosis^20^.

It is often difficult to distinguish the hypoplastic form of MDS (hMDS) from AA because both present with hypocellular BM. However, the risk of progression to acute leukemia is greater in hMDS, and differential diagnosis is important^21^. Although BM aspiration and biopsy examinations are essential to definite diagnosis, quantitative estimation of peripheral blood polymorphs including dysplastic features of granulocytes has been reported as a simple and valuable diagnostic tool in MDS^22^. Dysmorphic WBCs such as hypogranular neutrophils or pseudo-Pelger-Huet cells found in the PB are suggestive to differentiate hMDS from AA^23,24^.

Our work has several limitations: (1) although the accuracy of automated MDS diagnosis was over 90%, our system remains to be adjunctive in its nature since BM examination, clinical information, flow cytometric data, and genetic tests are essential for definite diagnoses of MDS^7^; (2) this was a single-center study with a relatively small number of samples, and the training sample patterns may have been incomplete; (3) we only used one combination of DLSs, CNNs, and XGBoost; and (4) while the infectious diseases were not studied in this study, it is important to distinguish MDS from AA with infection that can be accompanied with dysmorphic WBCs including toxic granulation, Döhle bodies and toxic vacuolation. In addition, other inflammation markers such as CRP are important to diagnose infectious diseases. Therefore, as a next step, we are planning to construct an advanced DLS trained with the extended data of serum biochemistry. It is indispensable to train this DLS with increased number of cases to cover various morphological changes of blood cells and to improve accuracy. Also, we are planning to develope a DLS to analyze images of BM samples.

The morphological approach continues to be fundamental at the beginning of the diagnostic algorithm, even the new molecular technologies including gene mutation and gene expression profiling are integrated with morphological examination in future^4,5^. Our approach might be applicable to develop new automated diagnostic systems for various hematological disorders.

## Materials and Methods

### Sample selection

The study has been approved by the the Juntendo University Hospital Medical Ethics Committee (Tokyo, Japan). As part of the approval, the ethics committee explicitly waived the need for informed consents from individual patients because all samples were de-identified in line with the Declaration of Helsinki. A total of 3,261 peripheral blood (PB) smears, including 1,165 from patients with hematological disorders, were prepared at Juntendo University Hospital (Tokyo, Japan) from 2017 to 2018. The slides were stained with May Grunwald-Giemsa using an SP-10 device (a fully automated slide-maker; Sysmex, Kobe, Japan). A total of 703,970 digitalized (preprocessed) cell images were collected with DI-60 automated digital cell image analyzer (Sysmex). The hematological disorders included MDS (n = 94), myeloproliferative neoplasms (n = 127), acute myeloid leukemia (n = 38), acute lymphoblastic leukemia (ALL, n = 27), malignant lymphoma (n = 324), multiple myeloma (n = 82) and AA (n = 42). Of all images, 695,030 were used to train the CNN-based image-recognition system, and 8,940 were used for validation. To develop an automated diagnostic system for MDS, 75 MDS and 36 AA cases were used for training. The gold standard of this study is the diagnosis by the hematopathologists in accordance with the latest guidelines^17^. All diagnoses were confirmed by independent hematopathologists based on clinical information, laboratory, flow cytometric, and genetic data, and BM aspiration and biopsy findings^25^.

### Data preparation

The training datasets were prepared for the recognition of image patterns by the deep learning system (DLS). The datasets were classified into 17 cell types and 97 abnormal morphological features by two laboratory technologists board-certified in hematology and one senior hematopathologist using the morphological criteria of the Clinical and Laboratory Standards Institute (CLSI) H20-A2 guideline and the 2016 revised WHO classification of myeloid neoplasms and acute leukemia^18^. After accumulating the image patterns using the training datasets, the performance of the DLS was evaluated using the validation datasets that were generated for testing the DLS by two laboratory technologists board-certified in hematology and one senior hematopathologist who are different from the ones worked on the training datasets. Table 1 summarizes the types and numbers of cell images used for training and testing.

### The deep convolutional neural network and training using individual cell images

**Figure 5:**
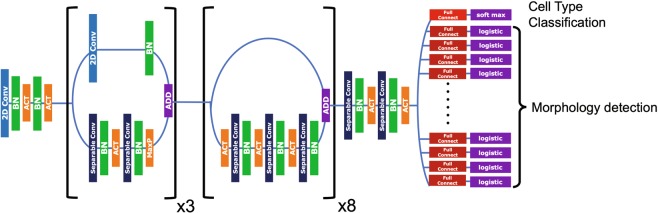
Schematic of the cell image-recognition system featuring convolutional neural networks. The deep learning system consists of three principal blocks, each of which contains piles of CNNs. The colors indicate different components of the CNNs.

To classify cells and identify morphological abnormalities simultaneously, we created a DLS-based cell image-recognition system composed of a CNN module that extracted features of preprocessed images and a classification module analyzing such features and classifying cell images into 17 cell types exhibiting some of 97 abnormal morphological characteristics (cell and nuclear size and shape, and cytoplasmic patterns). Figure 5 shows the overall structure of our image-recognition system. The “feature extraction module” is composed of two submodules. The first (upstream) submodule has three consecutive blocks, and each block follows two parallel pathways consisting of several convolutional network layers. These layer stacks optimize feature extraction from image data and output parameters to the next block. The second (downstream) submodule has eight consecutive blocks, each of which follows parallel pathways, one of which consists of a series of convolutional layers, whereas the other lacks convolutional components and is termed a residual network that functions as a buffer to avoid saturation of the system.

Each layer plays a different role: Separable Convolution (a specific type of convolutional layer; Conv 2D), Batch Normalization (BN), and Activation (ACT). Separable Convolution is a variant of regular convolution, in that spatial convolution is performed independently by each channel^26^. Conv 2D is a key component of neural networks that optimize parameters used to extract features and then processes the images to form “feature maps”^27,28^. BN normalizes input data distribution^29^. ACT follows, using a Rectified Linear Unit (ReLU)^30^. The first submodule was connected to the second to create feature maps. Conv 2D was bypassed in the second module to avoid unwanted deep layer saturation; this effectively calculates weights via back-propagation. The architecture was implemented using Keras^31^ and Tensorflow^32^.

### Extreme Gradient Boosting (EGB) to create a diagnostic algorithm for MDS

Next, we developed a system differentiating MDS from AA using cell image features extracted by the CNNs. To this end, we employed a XGBoost that uses a large ensemble of weak predictive models (such as decision trees) to recognize and classify the dysmorphic features/patterns of various blood cells^33^. XGBoost is one of the fastest and most efficient algorithms identifying optimal decision-making parameters^34^. First, we fed XGBoost with various cell image parameters, including the 17 cell classifications and 97 dysmorphic features identified by the CNN-based image-recognition algorithm. Then, we trained XGBoost using smears from the 75 MDS and 36 AA patients; XGBoost analyzed and remembered diagnostic cell patterns and dysmorphic features. Next, we used the 26 MDS and 11 AA samples to test the system. To determine how XGBoost made diagnostic decisions, the SHAP value of dysmorphic extents of various cell types were plotted on heat maps^35^.

## Supplementary information

A novel automated image analysis system using deep convolutional neural networks can assist to differentiate MDS and AA

## Data Availability

The datasets generated during and/or analysed during the current study are available from the corresponding author on reasonable request.

## References

[1] Bennett JM (1982). Proposals for the classification of the myelodysplastic syndromes. Br J Haematol.

[2] Cazzola M, Malcovati L (2005). Myelodysplastic syndromes–coping with ineffective hematopoiesis. N Engl J Med.

[3] Vardiman JW (2003). The new World Health Organization classification of myeloid neoplasms: Q&A with James W. Vardiman, MD. Clin Adv Hematol Oncol.

[4] Malcovati L (2014). Driver somatic mutations identify distinct disease entities within myeloid neoplasms with myelodysplasia. Blood.

[5] Veer Mvt, Haferlach T (2014). Should clinical hematologists put their microscopes on eBay?. Haematologica.

[6] Orazi, A., O’Malley, D. P. & Arber, D. A. *Illustrated Pathology of the Bone Marrow*. (Cambridge University Press, 2006).

[7] Brunning, R. D. B. *et al*. In *Pathology and Genetics of Tumours of Haematopoietic and Lymphoid Tis-sues*. 63–67 (IARC Press, 2001).

[8] Seo JY, Lee ST, Kim SH (2015). Performance evaluation of the new hematology analyzer Sysmex XN-series. Int J Lab Hematol.

[9] Cembrowski GS, Clarke G (2015). Quality control of automated cell counters. Clin Lab Med.

[10] Shafique S, Tehsin S (2018). Acute Lymphoblastic Leukemia Detection and Classification of Its Subtypes Using Pretrained Deep Convolutional Neural Networks. Technol Cancer Res Treat.

[11] Rehman A (2018). Classification of acute lymphoblastic leukemia using deep learning. Microsc Res Tech.

[12] MoradiAmin M, Memari A, Samadzadehaghdam N, Kermani S, Talebi A (2016). Computer aided detection and classification of acute lymphoblastic leukemia cell subtypes based on microscopic image analysis. Microsc Res Tech.

[13] Shafique S, Tehsin S (2018). Computer-Aided Diagnosis of Acute Lymphoblastic Leukaemia. Comput Math Methods Med.

[14] LeCun Y, Bengio Y, Hinton G (2015). Deep learning. Nature.

[15] Swerdlow, S. H. C. E. *et al*. *WHO Classification of Tumours of Haematopoietic and Lymphoid Tissues*. Revised Fourth Edition. (World Health Organization, 2017).

[16] Jamieson AR (2010). Exploring nonlinear feature space dimension reduction and data representation in breast Cadx with Laplacian eigenmaps and t-SNE. Med Phys.

[17] Killick SB (2016). Guidelines for the diagnosis and management of adult aplastic anaemia. Br J Haematol.

[18] Arber DA (2016). The 2016 revision to the World Health Organization classification of myeloid neoplasms and acute leukemia. Blood.

[19] Putzu L, Caocci G, Di Ruberto C (2014). Leucocyte classification for leukaemia detection using image processing techniques. Artif Intell Med.

[20] Esteva A (2017). Dermatologist-level classification of skin cancer with deep neural networks. Nature.

[21] Barrett J, Saunthararajah Y, Molldrem J (2000). Myelodysplastic syndrome and aplastic anemia: distinct entities or diseases linked by a common pathophysiology?. Semin Hematol.

[22] Hast R, Nilsson I, Widell S, Ost A (1989). Diagnostic significance of dysplastic features of peripheral blood polymorphs in myelodysplastic syndromes. Leuk Res.

[23] Mufti GJ, McLornan DP, van de Loosdrecht AA, Germing U, Hasserjian RP (2018). Diagnostic algorithm for lower-risk myelodysplastic syndromes. Leukemia.

[24] Bennett JM, Orazi A (2009). Diagnostic criteria to distinguish hypocellular acute myeloid leukemia from hypocellular myelodysplastic syndromes and aplastic anemia: recommendations for a standardized approach. Haematologica.

[25] Hong M, He G (2017). The 2016 Revision to the World Health Organization Classification of Myelodysplastic Syndromes. J Transl Int Med.

[26] Chollet, F. Xception: Deep Learning with Depthwise Separable Convolutions. *arXiv e-prints*, https://ui.adsabs.harvard.edu/ (2016).

[27] Goodfellow, I., Bengio, Y. & Courville, A. *Deep Learning*. (The MIT Press, 2016).

[28] Krizhevsky, A., Sutskever, I. & Hinton, G. E. In *Proceedings of the 25th International Conference on Neural Information Processing Systems* - Volume **1** 1097–1105 (Curran Associates Inc., Lake Tahoe, Nevada, 2012).

[29] Ioffe, S. & Szegedy, C. Batch Normalization: Accelerating Deep Network Training by Reducing Internal Covariate Shift. *arXiv e-prints*, https://ui.adsabs.harvard.edu (2015).

[30] Hahnloser RHR, Sarpeshkar R, Mahowald MA, Douglas RJ, Seung HS (2000). Digital selection and analogue amplification coexist in a cortex-inspired silicon circuit. Nature.

[31] Keras (https://keras.io 2015).

[32] TensorFlow: Large-Scale Machine Learning on Heterogeneous Systems (2015).

[33] Friedman JH (2001). Greedy function approximation: A gradient boosting machine. Ann. Statist..

[34] Chen, T. & Guestrin, C. In *Proceedings of the 22nd ACM SIGKDD International Conference on Knowledge Discovery and Data Mining*. 785–794 (ACM).

[35] Lundberg, S. M. & Lee, S.-I. In *Advances in Neural Information Processing Systems 30* (eds I. Guyon *et al*.) 4765–4774 (Curran Associates, Inc., 2017).

