# Supplementary material for: A novel automated image analysis system using deep convolutional neural networks can assist to differentiate MDS and AA
Source: Sci Rep. 2019 Sep 16;9:13385. doi: 10.1038/s41598-019-49942-z (PMC6746738; doi:10.1038/s41598-019-49942-z)
Supplement: Supplementary file 1 — A novel automated image analysis system using deep convolutional neural networks can assist to differentiate MDS and AA [file 41598_2019_49942_MOESM1_ESM.docx]

**TITLE**

**A novel automated image analysis system using deep convolutional neural networks can assist to differentiate MDS and AA**

**AUTHORS/AFFILIATIONS**

Konobu Kimura^1,2^, Yoko Tabe^1,3^, Tomohiko Ai^3^, Ikki Takehara^2^, Hiroshi Fukuda^4^, Hiromizu Takahashi^4^, Toshio Naito^4^, Norio Komatsu^5^, Kinya Uchihashi^2^, Akimichi Ohsaka^1,6^

^1^Department of Next Generation Hematology Laboratory Medicine, Juntendo University Graduate School of Medicine, Tokyo, Japan

^2^Sysmex Corporation, Kobe, Japan

^3^Department of Clinical Laboratory Medicine, Juntendo University Graduate School of Medicine, Tokyo, Japan

^4^Department of General Medicine, Juntendo University Graduate School of Medicine, Tokyo, Ja-pan

^5^Department of Hematology, Juntendo University Graduate School of Medicine, Tokyo, Japan

^6^Department of Transfusion Medicine and Stem Cell Regulation, Juntendo University Graduate School of Medicine, Tokyo, Japan

**Corresponding Author:** Yoko Tabe, M.D. Ph.D., Department of Next Generation Hematology Laboratory Medicine, Juntendo University Graduate School of Medicine 2-1-1, Hongo, Bunkyo-ku, Tokyo 113-8421 JAPAN

Phone: +81-3-3813-3111; Fax: +81-3-5684-1609; E-mail: tabe@juntendo.ac.jp

**SUPPROTING INFORMATION**

**Supplementary Figure S1. Representative images of dysmorphic peripheral blood cells.**


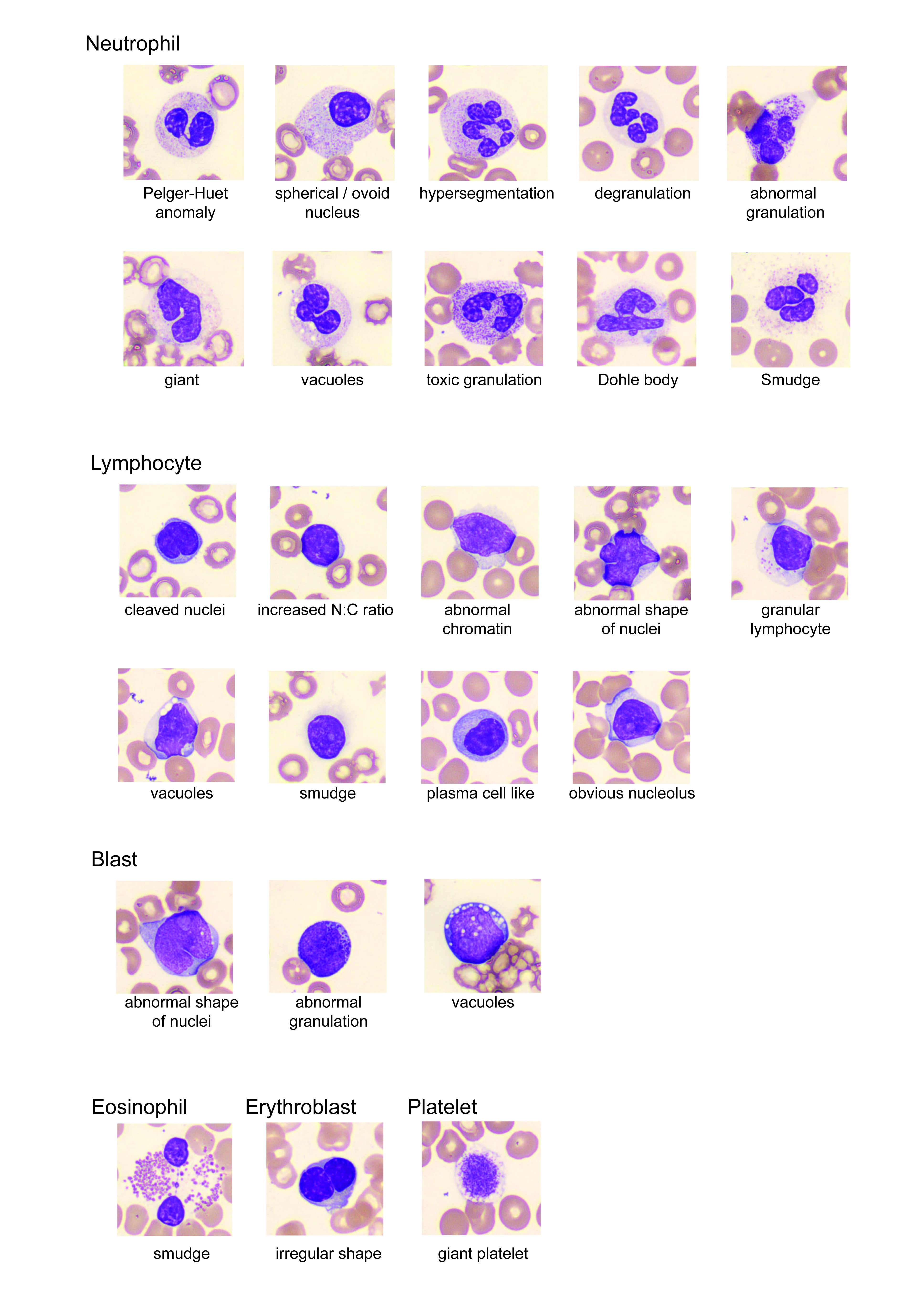


**Supplementary Table S1. Cell classification performance of the DI-60.**

| Cell type | Sensitivity (%) | Specificity (%) |
| --- | --- | --- |
| Segmented Neutrophil | 92.3% | 91.1% |
| Band Neutrophil | 26.7% | 98.9% |
| Metamyelocyte | 19.4% | 99.9% |
| Myelocyte | 58.9% | 99.5% |
| Promyelocyte | 75.7% | 99.4% |
| Blast | 80.0% | 98.8% |
| Lymphocyte | 81.8% | 93.6% |
| Variant Lymphocyte | 0.0% | 100.0% |
| Monocyte | 88.3% | 99.2% |
| Eosinophil | 72.1% | 99.9% |
| Basophil | 91.2% | 99.3% |
| Large Platelet | 98.1% | 99.3% |
| Megakaryocyte | 0.0% | 100.0% |
| Platelet Aggregation | 0.0% | 100.0% |
| Erythroblast | 92.4% | 99.6% |
| Smudge | 77.5% | 97.0% |
| Artifact | 61.7% | 99.3% |
